# Mixed Fortunes: Ancient Expansion and Recent Decline in Population Size of a Subtropical Montane Primate, the Arunachal Macaque *Macaca munzala*

**DOI:** 10.1371/journal.pone.0097061

**Published:** 2014-07-23

**Authors:** Debapriyo Chakraborty, Anindya Sinha, Uma Ramakrishnan

**Affiliations:** 1 Nature Conservation Foundation, Gokulam Park, Mysore, India; 2 National Centre for Biological Sciences, GKVK Campus, Bangalore, India; 3 National Institute of Advanced Studies, Indian Institute of Science Campus, Bangalore, India; Institut Pluridisciplinaire Hubert Curien, France

## Abstract

Quaternary glacial oscillations are known to have caused population size fluctuations in many temperate species. Species from subtropical and tropical regions are, however, considerably less studied, despite representing most of the biodiversity hotspots in the world including many highly threatened by anthropogenic activities such as hunting. These regions, consequently, pose a significant knowledge gap in terms of how their fauna have typically responded to past climatic changes. We studied an endangered primate, the Arunachal macaque *Macaca munzala*, from the subtropical southern edge of the Tibetan plateau, a part of the Eastern Himalaya biodiversity hotspot, also known to be highly threatened due to rampant hunting. We employed a 534 bp-long mitochondrial DNA sequence and 22 autosomal microsatellite loci to investigate the factors that have potentially shaped the demographic history of the species. Analysing the genetic data with traditional statistical methods and advance Bayesian inferential approaches, we demonstrate a limited effect of past glacial fluctuations on the demographic history of the species before the last glacial maximum, approximately 20,000 years ago. This was, however, immediately followed by a significant population expansion possibly due to warmer climatic conditions, approximately 15,000 years ago. These changes may thus represent an apparent balance between that displayed by the relatively climatically stable tropics and those of the more severe, temperate environments of the past. This study also draws attention to the possibility that a cold-tolerant species like the Arunachal macaque, which could withstand historical climate fluctuations and grow once the climate became conducive, may actually be extremely vulnerable to anthropogenic exploitation, as is perhaps indicated by its Holocene ca. 30-fold population decline, approximately 3,500 years ago. Our study thus provides a quantitative appraisal of these demographically important events, emphasising the ability to potentially infer the occurrence of two separate historical events from contemporary genetic data.

## Introduction

The Pleistocene epoch, with its frequent climatic fluctuations, is known to have driven range expansions and population decline/expansion of many species [1]. The climatic fluctuations consisted of episodes with relatively high and low global ice volumes. The lowest of ice volumes characterised the interglacial periods with relatively warmer climate [2]. The Last Glacial Maximum (LGM), which occurred near the end of the Pleistocene, about 20,000 years ago [3], was a particularly dramatic period of glacial advance and global cooling resulting in demographic bottlenecks for many animal populations. This period was eventually followed by a period of warm and humid climate that lasted through the Early Holocene period, with the subsequent expansion of many previously bottlenecked populations [4]. Importantly, the Holocene warming created opportunities for colonisation of new regions by modern humans as well, which further triggered profound alterations in world ecosystems and, in turn, the demography of several other species [5]. One of the ways we can corroborate the past occurrence of these large-scale changes is by tracking changes in the population size of species over evolutionary time.

Investigations into past complex dynamic events have been facilitated, in the absence of more accurate ancient DNA, by recent advances in model-based hypothesis testing of current molecular data. Thus, it is now possible to employ an inferential framework to derive statistically robust inferences of the timing and magnitude of past changes in the effective population size (*N*e) of species over clearly defined time periods [6], [7]. While it is crucial to identify and separate ancient from more recent demographic events to obtain insights into the long-term population dynamics of species, this is often difficult to achieve in practice as similar genetic patterns can result from different demographic histories [8]. Nevertheless, the use of different types of genetic markers, such as mitochondrial DNA and microsatellites, combined with new kinds of statistical analytical methods, have achieved significant success in detecting genetic signatures of major demographic events, such as population bottlenecks, expansions and admixtures that may have occurred in different historical time periods [6].

Although a vast majority of studies have performed these tests and estimated demographic parameters across taxa, very few studies have been conducted on tropical or subtropical ecosystems (but see [9], [10], [11]). This gap in our knowledge is clearly problematic as the detrimental effects of climatic and other environmental changes on subtropical and tropical biodiversity across much of the world are only likely to increase in the near future [12].

In order to address this knowledge gap, we sought to investigate the past demographic history of a primate, the Arunachal macaque *Macaca munzala*, an endangered species recently reported from and believed to be endemic to the state of Arunachal Pradesh in northeastern India. It is not at all clear how the complex climatic history of the Indian subcontinent might have shaped the population history of this primate, particularly given its distribution in the subtropical mountainous habitat within the Eastern Himalayan biodiversity hotspot.

Pleistocene climate change and/or physical barriers have often been described as important forces driving the demographic history of many species from other parts of the subtropics [13], [14], [15]. A few studies that have specifically examined the demographic history of primate species, however, suggest instead a primary role for anthropogenic factors driving relatively recent demographic changes in primate populations [9], [16], [17], [18]. The only reported exceptions to this trend are two macaque species from the northern hemisphere, the Barbary macaque *M. sylvanus* and the Japanese macaque *M. fuscata*. Their demographic history were shown to be shaped relatively more strongly by Pleistocene glaciation events than later anthropogenic influences [19], [20]. Consequently, these macaque populations revealed signatures of genetic bottlenecks after the LGM, around 20,000 years ago [19], [20]. We thus thought it important to explore the history of a macaque species in the Eastern Himalaya, which also marked the southernmost limit of the Pleistocene glaciers.

What also characterises this biodiversity-rich region is the decimation of its indigenous wildlife by the local human communities, a well-documented historical phenomenon. The state of Arunachal Pradesh is inhabited by a number of animistic tribes who continue to depend heavily on wildlife for their subsistence as well as for sport [21]. Our knowledge of the histories of these people is, however, fragmentary and incomplete. The peopling of this region seems to have occurred rather late in the history of the Indian subcontinent. According to variously recorded folklore and traditional knowledge, the ancestral Tani people migrated to central Arunachal Pradesh approximately 1,500 years ago from the north, through the Siang region. These people then settled in the Himalayan valleys and, due to the unique topography of the region as well as internecine warfare among them, were persistently isolated from one another. They were subsequently believed to have given rise to the many sub-tribes of today, such as the Adi and Apatani [22], which largely continue to follow their ancestral animistic culture. The Arunachal macaque is also known to be hunted across its distribution range, either in retaliation of its crop-raiding in the western districts of Tawang and West Kameng [23] or for food, sport and trade in the central districts of Upper Subansiri and West Siang [24].

In this study, therefore, we investigate the impact of two important factors, Pleistocene glaciation events and more recent anthropogenic activities, on the demographic history of the Arunachal macaque. More specifically, we ask whether the species was able to maintain its ancestral population size throughout its history on the southern subtropical fringes of the Tibetan Plateau. If, however, there were significant changes in the population size of this large mammalian species, were these wrought more by Pleistocene climate change, which is known to have affected the population history of several other species in the region, or were humans the principal driving force? Answers to these questions are critical not only to predict the future trajectories of populations of primates and other mammalian species to impending climatic changes in the Eastern Himalayan biodiversity hotspot but also to develop conservation strategies for the increasingly endangered species of the region.

## Methods

### Ethics statement

This study was conducted in accordance with all relevant Indian laws, with due permits from the State Forest Department of Arunachal Pradesh. We collected only small pieces of dried skin samples from local communities without any kind of payment. As these were from old hunting trophies and no fresh hunting was reported from our study sites, we are confident that our sample collection did not encourage killing of the species in any way. Additionally, we have led a conservation programme for the species in this region over the last seven years.

### Study area and population sampling

We obtained dried skin samples of Arunachal macaque individuals, killed and preserved as hunting trophies, from 14 villages across the districts of Tawang, Upper Subansiri and West Siang in the state of Arunachal Pradesh (Fig. S1). While Tawang forms the western edge of the state, both Upper Subansiri and West Siang are located in remote central Arunachal Pradesh. These samples were preserved in 95% ethanol at ambient temperature till they were transported to the laboratory. A single blood sample was also obtained from a captive individual in Typee, Tawang District. We considered the samples to belong to three tentative populations – Tawang (n = 5), Upper Subansiri (n = 12) and West Siang (n = 9) on the basis of their geographic origins (Table S1).

### DNA extraction and PCR amplification

We extracted genomic DNA from skin and blood samples using the QIAGEN DNeasy Blood & Tissue Kit (Qiagen GMBH, Hamburg, Germany), following the manufacturer's protocols. We used extraction blanks as negative controls in downstream polymerase chain reaction (PCR) amplifications.

In order to sample a non-coding region of the Arunachal macaque mitochondrial genome, we amplified a 534bp-long D-loop (hyper-variable segment 1) region using the primer set from Li and Zhang [25]. We conducted standard 35-cycle PCR to amplify the target regions [26].

We amplified 22 fluorescently labelled microsatellite loci (DXS571, DXS6810, DXS8043, DXS6799, D20S171, D4S243, D12S372, D8S1466, D9S934, D7S794, D10S611, D8S1106, D15S823, D19S255, D2S146, D17S791, D18S869, D18S537, D6S2419, D16S403, D5S1457, D10S179, D11S2002), already established for other macaque species [27], [28]. PCR amplification of 35 cycles was conducted for up to five loci simultaneously with combinations selected on the basis of fragment size, annealing temperature, and the fluorescent dye set DS - 33 components used (dy6FAM, VIC, PET or NED). The PCR products were resolved with an ABI 3130xl automated sequencer and analysed with GeneMapper software (version 4.0; Applied Biosystems, Foster City, USA). We could successfully sequence 24 of the individual samples collected – 5 for Tawang, 10 for Upper Subansiri and 9 for West Siang. The accession numbers for the mitochondrial DNA sequences have been given in Table S1. We are also open to sharing the microsatellite data for further analyses, if requested.

### Population demographic history

#### Bayesian skyline plots

We used Bayesian skyline plots of mitochondrial DNA (mtDNA) to examine the past population dynamics of the study populations.

Bayesian skyline plots allow for the estimation of effective population size, *Ne*, through time without specifying population change models such as constant size or exponential growth. The age of ‘the most recent common ancestor' or TMRCA of each clade [29] – Tawang: 0.12 mya (95% Highest Posterior Density or HPD 0.05 – 0.21), Upper Subansiri: 0.46 mya (95% HPD 0.27 – 0.68) and West Siang: 0.89 mya (95% HPD 0.53 – 1.3) – were employed as priors for the three populations. Furthermore, we selected the HKY+Γ+I mutation model for the analysis with a mean HVS1 substitution rate of 0.1643 substitutions per nucleotide per million year (Myr) for the mean rate prior [29]. We used BEAST, version 1.7 [30] for constructing the Bayesian skyline plots. Two independent runs were conducted for 10^7^ steps, with a sampling of the parameters once every 10^3^ steps. Ten percent of the samples were discarded as burn-in. The estimated sample size (ESS) for all parameters was found to be above 200 at the end of each run, indicating good convergence of the Markov chain Monte Carlo (MCMC) simulations. The independent runs were combined using LogCombiner, version 1.5.4 [30], with resampling every 10^5^ steps. After combining all the MCMC runs, the ESS for all parameters was observed to be more than 200.

#### The EWCL (Ewens, Watterson, Cornuet and Luikart) method

Demographic events such as recent population bottlenecks are known to leave distinct genetic signatures in the distributions of allele size, expected heterozygosity and in the genealogy of microsatellite loci [33], [9]. Here, we used the allelic frequency spectrum, namely the number of alleles *(n_A_)* and the expected heterozygosity *(H_e_)*, to determine the patterns of genetic diversity expected for a demographically stable population [32]. We performed simulations in Bottleneck, version 1.2.02 [33], for both the species separately, to obtain the distribution of *H_e_*, conditional on *n_A_* and on the sample size for each population and locus. These *H_e_* values were then compared to those obtained from the real dataset. Three mutation models were used: the infinite-allele model (IAM), the stepwise-mutation model (SMM) and the two-phase model (TPM), with various amounts (70% to 95%) of single-step mutations [34]. Any departures from the null hypothesis were explained as departures from the model, including selection, population expansion or decline. Consistency across independent loci, however, was unlikely to be caused by selection but rather by demographic events. This approach allowed us to detect population size changes and confirm that the signal was consistent across mutation models. The demographic event, however, could not be dated.

#### The Approximate Bayesian Computation approach

We further inferred the demographic history of the Arunachal macaque, based on microsatellite data, using the approximate Bayesian computation (ABC) approach [35], implemented in the programme DIY-ABC, version 1.0.4.46b [36]. This approach allowed us to choose a demographic scenario among many that best fits the data and infer the posterior probability distributions for the parameters of interest under this preferred scenario. The different steps of the ABC parameter estimation procedure [37] are briefly described here.

**Figure 1:**
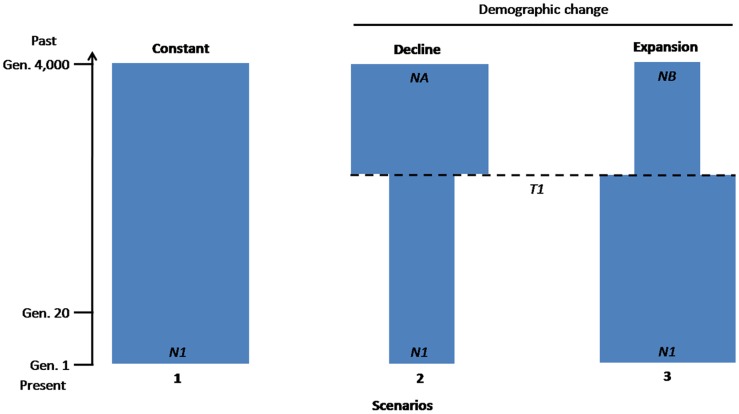
Possible alternative scenarios of the demographic history of the Upper Subansiri population. When tested using the ABC approach, Scenario 2 was best fit with the data (Table 2). The details of each scenario parameterisation have been given in the Methods. The time-scale is indicated by the arrow on the left. T*_1_* ranges between 20 and 4000 generations. Time has been measured backward in generations before the present. Gen: Generation.

We compared three demographic scenarios, graphically depicted in Fig. 1. Scenario 1 consisted of a null hypothesis that assumed a population whose effective size (*N_1_*) remained stable over time. Scenario 2 assumed a population of size *N_A_* that declined instantaneously to its current effective size (*N_1_*), *T_1_* generations ago. Conversely, Scenario 3 assumed a population of effective size *N_B_* that increased instantaneously *T_1_* generations ago to reach its current effective size (*N_1_*). For Scenarios 2 and 3, we considered *T_1_* to be between 20 and 4000 generations, during which time these events could have occurred.

For each of the three models, we simulated one million datasets based on a demographic history that described the model, using the programme DIY-ABC. Some or all parameters that defined each model (such as population sizes, timing of the demographic events or mutation rates) were considered as random variables for which some prior distributions were defined, as shown in Table 1. For each simulation, the parameter values were drawn from their prior distributions, defining a demographic history that was used to build a specific input file for the DIY-ABC programme. Coalescent-based simulations were run to generate a genetic diversity for each sample, with the same number of gene copies and loci as those originally observed. Summary statistics (S) were then computed for the simulated datasets for each of the observed dataset (S*). Following the method of [38], a Euclidean distance, δ, was calculated between the normalised S and S* for each simulated dataset.

**Table 1:** Model specifications and prior distributions for demographic parameters and locus-specific mutation model parameters.

Priors for the Demographic Parameters		
*N_1_*		UN ∼ [50, 5000]
*T_1_*		UN ∼ [20, 4000]
*N_A_*		UN ∼ [10000, 70000]
*N_B_*		UN ∼ [10, 5000]
**Priors for the Mutation Model**		
Autosomal microsatellites		
MEAN – *μ_mic_*	UN∼[1×10^−4^, 1×10^−3^]	
GAM – *μ_mic_*	GA∼[1×10^−5^, 1×10^−2^, 2]	
MEAN – *P*	UN∼[0.10, 0.30]	
GAM – *P*	GA∼[0.01, 0.9, 2]	
MEAN – *SNI*	LU∼[1×10^−8^, 1×10^−4^]	
GAM – *SNI*	GA∼[1×10^−9^, 1×10^−3^, 2]	

**Figure 2:**
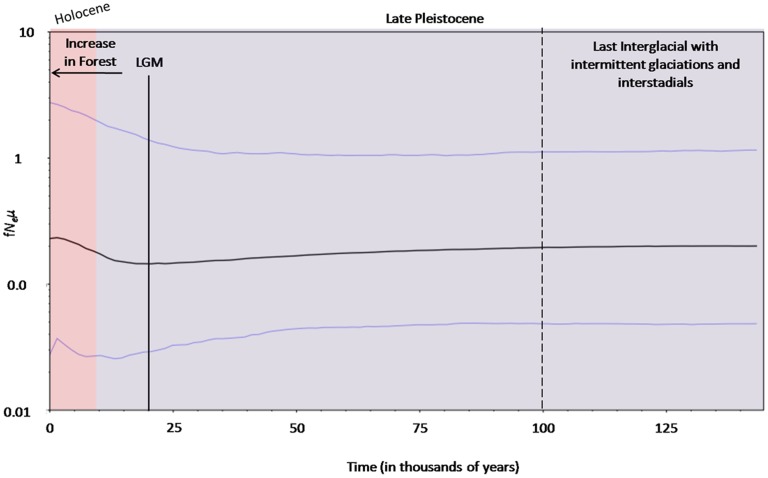
Bayesian skyline plot reconstruction of past population size trajectory for the Upper Subansiri population. The plot is the product of female effective population size (f*N_e_*) and mutation rate (*μ*) through time, assuming a substitution rate of 0.1643 substitutions per nucleotide per million years [29]. The lower and upper 95% confidence interval for the Upper Subansiri TMRCA is also shown. LGM, Last Glacial Maximum, approximately 18 to 20 thousand years before present.

UN: Uniform distribution, with two parameters – minimum and maximum values; GA: Gamma distribution with three parameters – minimum and maximum values and shape parameter value; LU: Log-Uniform distribution with two parameters – minimum and maximum values. See Figure 2 for the demographic parameters of each model tested. The mutation model parameters for the microsatellite loci were the mutation rate (*μ_mic_*), the parameter determining the shape of the gamma distribution of individual loci mutation rate (*P*), and the Single Insertion Nucleotide rate (*SNI*).

The 22 microsatellite loci were assumed to follow a generalised stepwise mutation model [39] with two parameters: the mean mutation rate (*μ_mic_*) and the mean parameter of the geometrical distribution assumed for the length in repeat numbers of mutation events (*P*) drawn from uniform prior distributions of 10^−4^ to 10^−3^ and 0.1 to 0.3, respectively. Each locus has a possible range of 40 contiguous allelic states, except Locus 12 (43 states) and was characterised by individual *μ_loc_* and *P_loc_* values, drawn from gamma distributions with respective means of *μ_mic_* and *P*, and shape parameter 2 in both cases [40]. Using this setting, we allowed for large mutation rate variance across loci (range of 10^−5^ to 10^−2^). We also considered mutations that insert a single nucleotide into or delete one from the microsatellite sequence. We used default values for all other mutation model settings. The details on model parameterisation and prior settings have been provided in Table 1.

The summary statistics of genetic diversity for the microsatellite loci, calculated with the programme DIY-ABC, included: the mean number of alleles per locus (*A*), mean expected heterozygosity (*H_e_*), mean allele size variance (*V*), and mean GW Index across the loci [31] (Garza and Williamson 2001).

The posterior probability of each competing scenario was estimated using a polytomous logistic regression [41], [36] on 1% of the simulated datasets closest to the observed dataset. We evaluated the ability of our ABC methodology to discriminate between scenarios by analysing simulated datasets with the same number of loci and individuals as in our real dataset. Following the method of Cornuet *et al*. [36], we estimated the Type I error probability as the proportion of instances where the selected scenario did not exhibit the highest posterior probability, as compared to the competing scenarios for 500 simulated datasets generated under the best-supported model. We similarly estimated the Type II error probability by simulating 100 datasets for each of two alternative scenarios and calculating the mean proportion of instances in which the best-supported model was incorrectly selected as the most probable model.

We estimated the posterior distributions of the demographic parameters under the best demographic model, using a local linear regression on the closest 1% of 10^6^ simulated datasets, after the application of a logit transformation, the inverse of the logistic function, to the parameter values [35], [41]. Finally, following the method of Gelman *et al.* [42], we evaluated whether, under the best model-posterior combination, we were able to reproduce the observed data using the model-checking procedure available in DIY-ABC [36]. Model-checking computations were processed by simulating 1000 pseudo-observed datasets under each studied model-posterior combination, with sets of parameter values drawn with replacement among the 1000 sets of the posterior sample. This generated a posterior cumulative distribution function for each summary statistic, allowing us to estimate the *P* values for the observed values of these summary statistics. In addition, a principal components analysis (PCA) was performed on the summary statistics. Principal components were computed from the 15,000 datasets simulated with parameter values drawn from the prior. The target (observed) dataset, as well as the 1,000 datasets simulated from the posterior distributions of parameters, was then added to each plane of the PCA.

## Results

### Bayesian skyline plots

Our mitochondrial DNA analysis (see also [29]) demonstrated that the three Arunachal macaque populations, Tawang, Upper Subansiri and West Siang, are geographically distinct. Macaque populations, being female philopatric, tend to exhibit such distinctive population genetic structure for maternally inherited mitochondrial DNA [19], [20]. Given this population structure, we treated each population separately for demographic analyses. Conducting a skyline plot with data from all three populations combined would result in a putatively incorrect signature of population expansion since these population show significant structure [8]. Of these three populations, the Upper Subansiri had the largest number of sampled individuals and was, therefore, taken up for further analysis. It must be noted, however, that we could not rule out the existence of local migrants and their influence on our results, as we could not sample all the populations in the intervening time periods.

The Bayesian skyline plot for the Upper Subansiri population indicated a population expansion right after the Last Glacial Maximum (LGM) at around 15,000 years before present (Fig. 2). The population, however, appeared to have maintained a constant population size before this expansion. The plot also revealed a small reduction in effective population size in more recent times, right after the Middle Holocene (between 5,000 and 7,000 years before present; Fig. 2), an indication that the distribution may have stabilised around the mean, given that there is no detectable change in trends for the upper bound. Finally, it must be noted that such horizontal distribution can also be drawn within the credibility interval and, as a result, the mean effect may represent a simple trend rather than true statistical evidence of actual demographic shifts.

### The EWCL (Ewens, Watterson, Cornuet and Luikart) method

This test, which attempted to detect the presence of a population bottleneck, indicated that, regardless of the mutation model assumed, the three Arunachal macaque populations, taken together, exhibited a significant signal of bottleneck for a majority of the microsatellite loci tested. It should, however, be remembered that this can be an artefact of the presence of structure in the tested populations. We, therefore, decided to treat the populations separately and found that only the Upper Subansiri site had an acceptable sample size for the test. For this population, the infinite-allele model (IAM) and two-phase model (TPM) showed statistical significance for heterozygosity excess, in both one-tailed and two-tailed tests (both *P* << 0.05), thus providing clear evidence for a genetic bottleneck in this population in the past. The application of a stepwise-mutation model (SMM), however, yielded non-significant results for both one-tailed (*P* = 0.13) and two-tailed (*P* = 0.064) tests.

### The Approximate Bayesian Computation approach

**Figure 3:**
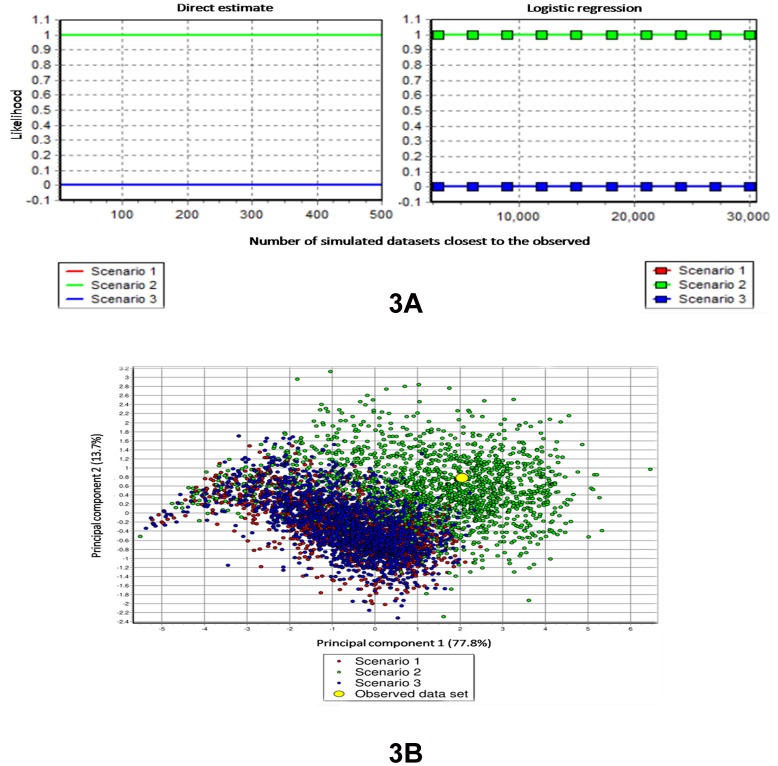
Comparison of the three possible alternative demographic scenarios for the Upper Subansiri population. (A) Estimates of the posterior probability of each scenario and their comparison. Direct estimate: The number of times that a given scenario is found closest to the simulated datasets once the latter, produced under several scenarios, have been sorted by ascending distances to the observed dataset. Logistic regression: A polytomic weighted logistic regression was performed on the first closest datasets with the proportion contributed by each scenario as the dependent variable and the differences between the summary statistics of the observed and simulated datasets as the independent variables. The intercept of the regression, corresponding to an identity between simulated and observed summary statistics, was taken as the point estimate. In addition, 95% confidence intervals were computed [41]. In all these cases, Scenario 2 explained the observed data best. (B) Principal component analysis: Visual information on how data sets simulated under each scenario are close to the observed data set. Here too, Scenario 2 fits the observed data best [41].

We then tested three alternative scenarios of demographic change – demographic expansion, decline or constant size – using a model-based approximate Bayesian inferential framework. We first evaluated the relative posterior probability of each competing scenario using a polytomous logistic regression on 1% of the simulated datasets closest to the observed dataset. The resulting PCA unambiguously pointed to the scenario of population decline (Scenario 2), which assumed an ancient, large population size that declined at some point in the past to reach the present, much smaller population size (Fig. 3).

We next evaluated the power of the model choice procedure using the method implemented in DIY-ABC, following the recommendations of Robert *et al.* [43]. For this purpose, we first simulated 500 random datasets under the selected scenario (Scenario 2) and computed the proportion of cases in which this scenario did not display the highest posterior probability among all scenarios. This empirical estimate of the Type I error was only 16.6%. We then empirically estimated the Type II error rate by simulating 100 random datasets under each alternative scenario (Scenarios 1 and 3) and computing the proportion of cases in which Scenario 2 was incorrectly selected as the most likely scenario in these simulated datasets. The average Type II error rate was only 8%, indicating a statistical strength of 92%. Hence, this simulation-based evaluation of the performance of the ABC model-choice procedure [43] clearly showed that, given the size and polymorphism of our dataset, the method had statistical properties of both power and robustness to distinguish between the alternative demographic scenarios that we investigated.

We estimated the marginal posterior probability density for each parameter of the decline scenario (Scenario 2) using 1% of the closest simulated datasets from the observed dataset (Table 2). Under this model, we estimated that a large population with an effective size of approximately 50,600 macaque individuals (95% Highest Probability Density or HPD 19,600 – 68,300) declined to a present effective population size of approximately 1,700 individuals (95% HPD 476 – 4,090), that is, approximately 30-fold. Assuming an average generation time of five years for macaques [44], we estimated that this decline occurred approximately 3,500 (95% HPD 520 – 13,800) years before present.

**Table 2:** Demographic parameters estimated under the best-supported demographic scenario (Scenario 2) of a recent population decline in the Upper Subansiri population of Arunachal macaques.

Parameter	Median	25% HPD	95% HPD
Current effective population size (*N_1_*)	1720	476	4090
Population size before the decline (*N_A_*)	50600	19600	68300
Magnitude of the population decline (*N_1_*/*N_A_*)	0.03	0.02	0.06
Time since the decline (*T_1_*), years before present	3575	520	13800

Population sizes are given in effective number of diploid individuals. Time estimates were calibrated by assuming a generation time of 5 years [44].

**Figure 4:**
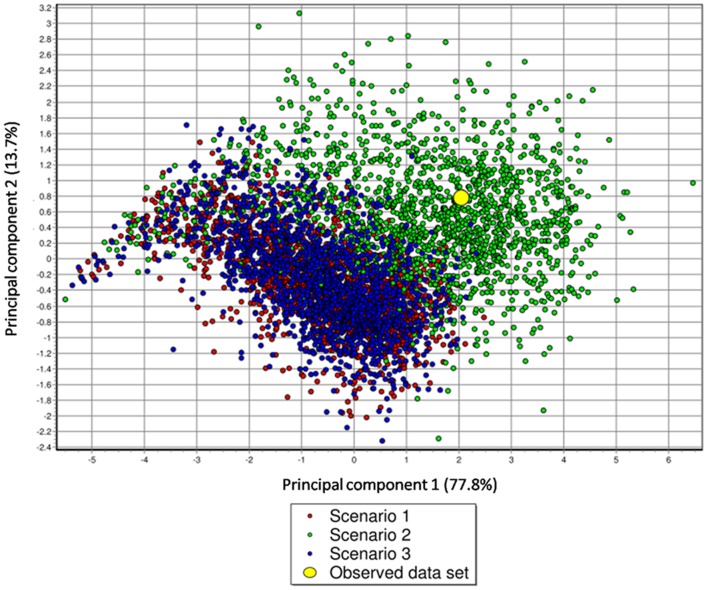
Model-checking to measure the discrepancy between the model parameter posterior combination and the real dataset for the three alternative demographic scenarios for the Upper Subansiri population of the Arunachal macaque. The summary statistics using datasets simulated with the prior distributions of the parameters, the observed data and the datasets from the posterior predictive distributions are represented on the plane of the first two principal components. The model (Scenario 2) fits the data well, as the cloud of datasets simulated from the prior (small green dots), datasets from the posterior predictive distribution (larger green dots) and the observed dataset (yellow circles) overlap completely.

Finally, to assess the goodness-of-fit of the model (Scenario 2) to the data, we simulated 1,000 datasets under each of the three scenarios tested, drawing the values of their parameters into the marginal posterior distributions of these parameters. We thus identified which model was the most capable of reproducing the observed summary statistics computed from the real data, following the model-checking procedure described by Cornuet *et al.* [36]. Of the three demographic scenarios tested, the datasets simulated under Scenario 2 were most compatible with the observed summary statistics. This can be observed by comparing the values of summary statistics computed from the simulated datasets for each tested scenario against the real values. With the exception of Scenario 2, all competing scenarios generated large numbers of summary statistics that displayed highly significant outlying values (Fig. 4). This finding thus strongly supported the very high posterior probability values obtained for Scenario 2 of population decline, using the model-choice procedure.

## Discussion

Classical bottleneck tests are often used by both evolutionary and conservation biologists to evaluate whether species have experienced historical demographic declines. In contrast, more recently developed Bayesian approaches offer the potential to draw more detailed inferences with respect to both bottleneck timing and severity [35], [36] but have not yet been thoroughly evaluated. Consequently, we analysed a multi-marker dataset using both sets of approaches to elucidate the possible impacts of historical climate change and more recent anthropogenic effects on an “edge” primate species – a species that inhabits the undulating edges of a plateau instead of its flat centre [14] – the Arunachal macaque, in the northeastern Indian state of Arunachal Pradesh. This region, situated in the southern part of the Tibetan Plateau, also forms part of the Eastern Himalaya biodiversity hotspot.

### Limited effect of Pleistocene glaciations before the Last Glacial Maximum (LGM)

Pleistocene climate change is known to have influenced the demographic history of many species across a wide variety of taxa. The landmass of Europe and a large part of northern America were under continental ice sheets repeatedly over the last three million years, forcing most species to either go extinct or shift their distribution range. Those, which shifted their range, also suffered a concomitant reduction in their population size. The effects of glaciation on species abundance and distribution, however, become more complex in eastern Eurasia where ice cover was never continuous [45], [46].

The southern part of the Tibetan Plateau is an interesting example in this regard. The southern edge of the plateau was characterised by complex orogenesis, as compared to that experienced by the flatter central regions. This area also marks the southern limit of the glaciers that occur on the plateau. During the Quaternary period, the Tibetan Plateau had undergone four to five glaciations but was less affected by the ice sheets than were other regions in Asia at that time [47]. The largest glacier on the plateau occurred in the middle of the Pleistocene (about 0.5 mya) and continued until 0.17 mya [47]. During that time, the ice cover may have been permanent in the higher altitudes and central regions of the plateau [48], [49] while the southern and eastern regions experienced relatively less glaciation [50]. Both these sets of conditions created a fragmented ice cover where most of the high-elevation areas on the mountain ranges were ice-clad. Consequently, the prolonged glaciation periods during the late Pleistocene appear to have had a less severe effect on the species inhabiting these areas, as compared to those inhabiting Europe or North America.

It is now being suggested that the ice sheets possibly retreated more rapidly on the Tibetan Plateau than they did in Europe, as seems to be evident from the demographic history of species like the great tit *Parus major* from the plateau [46]. It was observed that contrary to the post-LGM expansion of European animal populations, the demographic histories of species on the Tibetan Plateau indicate expansions only before the LGM; they remained relatively stable or grew slowly subsequently through the LGM [46]. The edges of the plateau would have been less under the influence of the glaciations, if at all, at least on the lower elevations. We find support for such a hypothesis in the Bayesian skyline plots, which suggest that the size of the Arunachal macaque populations appeared to remain constant during the prolonged pre-LGM climatic fluctuations. A recent comparative study on five avian species from the Tibetan Plateau [14] demonstrated that three species distributed on the central platform of the plateau experienced rapid population expansion after the retreat of the extensive glaciers during the pre-LGM (0.5–0.175 mya), results similar to that of Zhao *et al*. [46]. On the contrary, the population sizes of the other two species, the twite *Carduelis flavirostris* and the black redstart *Phoenicurus ochruros*, distributed on the edges of the plateau, remained at stable levels throughout the same pre-LGM period. It was concluded that the comparatively ice-free habitats on the edges of the plateau might have experienced milder climates during the glaciation period and this allowed the local species populations to persist in this stable niche [14]. Our data also allow us to arrive at similar conclusions for a mammalian species, the Arunachal macaque, in the Eastern Himalayas on the southern fringes of the Tibetan Plateau.

### Effect of elevation and animal physiology

Altitude appears to have played an extremely important role in the demographic history of animal species during the past glacial periods with species at various elevations being affected differently. A very good example of this is in the Alps [51], [52]. Many present-day species in the high elevations colonised their present range by expanding their range from the lower altitudes and latitudes during specific interglacial periods. In contrast, species with Arctic–Alpine distributions, which were not annihilated during peaks of glaciation, descended during these periods and may have spread more widely across the cold tundra and steppe plains of continental Europe [51], [52]. The impact of Pleistocene glacial cycles is thus expected to have varied among species across geographical regions, in part, perhaps due to their biology of differential cold tolerance [15].

The first indication of such a historical change in the size of the study Arunachal macaque populations was detected through the mismatch distributions of the sampled individuals (data not shown) which, however, could be unreliable due to small sample size of the present study. Mismatch distribution analysis also has its limitations, as exemplified by many studies where contemporary samples of a bottlenecked population failed to recover a unimodal mismatch distribution [53]. Instead, coalescent theory, which incorporates information from genealogy may better able to evaluate the demographic histories of populations [54], [55]. Bayesian skyline plots in our study also suggest expansion of the study macaque populations. These plots also propound changes in population size only after the LGM, as has been established for several avian edge species on the Tibetan Plateau [14]. Moreover, the effective female population size of the Arunachal macaque seems to have increased approximately 15,000 years ago.

Although the climate on the Tibetan Plateau has been documented to have been colder during the pre-LGM extensive glacial period than during the LGM [56], mountain heights below the snowline were not apparently glaciated. The current snowlines on the eastern edge of the plateau extend from 4200 to 5200 m [57], [58]. In Arunachal Pradesh, more specifically, the vegetation ends approximately at a 5,000-m elevation from where the present snowline starts [59]. During the LGM, however, the snowline descended to approximately 3,300 m on many mountain ranges on the Tibetan Plateau [56], [57], thus contributing to cooler climates locally [47]. Arunachal macaques are known to presently occur at altitudes between 1800 – 3500 m [60] although they could also inhabit higher, unexplored, elevations in the region. It now seems possible that at least a part of the current habitat of the macaque was covered by ice during the LGM. We, therefore, speculate that this primate might have colonised even higher elevations after the LGM, resulting in the observed signature of population expansion.

The pattern that our results suggest have been conclusively established for another lower-elevation avian edge species from the Tibetan plateau, the black redstart *Phoenicurus ochruros* [14]. The relationship between glaciation, altitude and species demographic history is, however, far from straightforward. Interestingly, Lu *et al*. [15] found that the population size of the three low-elevation (1800 – 3200 m) stream salamander species from the Tibetan Plateau were significantly but slowly decreased from the beginning of the extensive glacial period long before the LGM, in striking contrast to what has been established for the Arunachal macaque and the black redstart. According to Lu *et al.* [15], the suitable habitats for the salamanders may have not been covered by ice during both the LGM and the extensive pre-LGM glacial period but the species may have suffered from climatic cooling because they are less cold-tolerant than their high-elevation counterparts. These ideas are consistent with the hypothesis that variation in ecological adaptations may affect geographical patterns of genetic variation in species populations [61], [62]. The poikilothermic salamanders may be expected to have a much lower range of tolerance to changes in ambient temperatures than would homoeothermic animals such as birds and mammals. Thus, animal physiology is another important factor that may have differently affected the evolutionary history of species that otherwise occurred at similar elevations and latitudes in a particular geographical region.

### Holocene population decline

The increase in effective female population size of the Arunachal macaque, as revealed by the Bayesian skyline plots, continues till approximately 5,000 years ago, after which it appears to have suffered a mild decline. Mitochondrial and nuclear autosomal microsatellite loci are known to be informative at different time scales, highlighting different episodes in the demographic history of a species [6]. The mtDNA diversity tends to reflect comparatively older demographic events in a species' history. Conversely, microsatellite data are more informative about the contemporary demography of a species. Such a difference in demographic signals, captured by each type of genetic marker, may arise from the differences in their respective mutation rates [36]. The relatively fast mutation rate of microsatellite loci enables them to capture recent and almost contemporaneous events but also increases homoplasy at these loci, which thereby reduces the signal of older demographic events [39]. These, more ancient, events can, however, still be detected using the slower evolving mtDNA sequences. That is why we employed our microsatellite data to validate the occurrence of a very recent population decline in the Arunachal macaque.

The classical heterozygosity excess test (the EWCL method) suggested a past population bottleneck in at least the Upper Subansiri population, as reflected in its microsatellite data although the statistical significance of this result was highly dependent on the underlying mutational model. A bottleneck was inferred for this population for two particular models – the infinite-allele (IAM) and two-phase (TPM) models – but not for a third one, the stepwise-mutation (SMM) model, despite the IAM being unrealistic for most microsatellites [34]. Several other microsatellite-based studies of species, thought to have experienced severe, but temporary, reductions in population size, had either failed altogether to detect a bottleneck or, as with our study, yielded results that depended on the mutational model [63]. In at least some of these cases, a few specific markers may have disproportionately influenced the more conservative SMM model. However, in our study, we used 22 microsatellite loci, over twice the minimum number recommended [64]. To circumvent this problem, we applied the ABC method that clearly supported the population decline model (Scenario 2) over the constant size and population expansion models.

We could also estimate the extent and time of the decline from the posterior probabilities. Our simulations suggest the population decline started approximately 3,500 years ago, which was climatically a warm period and this is surprising as such climatic conditions are normally conducive for population growth. Several authors have postulated that such idiosyncratic mid-Holocene population declines may have been accelerated and enhanced by major expansions in ancient human civilizations [65] (ca. 1,500 BC), the rapid development of agriculture, and the resulting changes in landscapes in recent times. For example, it has been speculated that the expansion and migration of human populations into the erstwhile virgin mountains of the Sichuan region may have caused the decline of the giant panda population during the later part of the mid-Holocene [66].

In Arunachal Pradesh too, anthropogenic factors may have significantly influenced the demographic history of the Arunachal macaque over the last few decades or centuries. One of the largest indigenous groups of people that inhabit the districts of Upper Subansiri and West Siang, two areas from which our study samples were derived, and which hunt wildlife extensively for food and sport, is represented by the collective animist Adi people [67], [68]. Recent ethnographic and population genetic studies reveal that the Adi, alternatively referred to as the Luoba Tibetan in Tibet, trace their ancestral migration and settlement history from southern Tibet into Arunachal Pradesh to different time periods during the 5^th^ to 7^th^ century AD, or to the last 1,300 to 1,500 years before present [67], [68], [69], [70]. Although these estimates do not reject the possibility of a peopling of this region in even earlier times, it is entirely possible that the Arunachal macaque population declines may have actually begun at the time that central Arunachal Pradesh was being peopled by the Adi and other animistic tribes that continue to hunt rampantly even today. It must be reiterated, however, that there is currently no evidence to conclusively establish the definitive contribution of either climatic or anthropogenic factors to this decline in Arunachal macaque populations.

It should be noted that our investigation, which focuses on a rare, endangered, montane primate, has a smaller sample size than is typical for most population genetics studies. Although there appears to be little benefit in sampling more than 20 to 30 individuals per population for microsatellite-based studies that assess genetic diversity of populations [71], [72], sample sizes that are even smaller, as is the case in the present study, may not precisely represent the populations and may yield large errors for descriptive statistical estimates such as allele frequencies and expected heterozygosity. It has, however, been suggested that where small sample sizes are unavoidable, as, for instance, in the case of rare and endangered species, the precision of the estimates can be significantly enhanced by increasing the number of genotyped microsatellite loci, which is precisely what we have done in our study [72]. Moreover, we have used approximate Bayesian computations to test whether the population size changes that we observe using other analytical methods are noteworthy or not. The approximate Bayesian computation framework manages any sample size, which may often be as low as 8 – 12 samples per population [73], by simulating the exactly observed sample sizes [74]. Small sample sizes would simply lead to wider credible intervals than would larger sample sizes [74]. We are aware of this limitation of our results but believe that the communication of our present study is of critical importance, in spite of its low sample size, for the conservation effort to save the species, particularly as the collection of more samples from the rare and heavily hunted macaque populations in Arunachal Pradesh is not currently feasible.

In conclusion, the Tibetan Plateau is an important region to study the population history of its native species for more than one reason. First of all, it is a highly variable region with its different regions – the flat central platform and the more variable mountainous edges based on altitude – differing significantly from one another in topography and climatic history. The edge regions of the plateau have had a tumultuous orogenic history and the species of these areas are, thus, also expected to show a more eventful evolutionary history. Our study of an edge primate species has, along with other studies from the region, hinted at a very complex image of how geographical features such as latitude and altitude, animal physiology, and climate change in the past may have together influenced its demographic history. Unlike in the tropics, where Pleistocene glacial fluctuations were less likely to have been extreme, this region seems to represent a balance between the tropics and the more severe, temperate arctic environments. This study also underscores the possibility that a cold-tolerant species like the Arunachal macaque, which could withstand historical climate change and grow once the climate became conducive, may actually be extremely vulnerable to anthropogenic exploitation, as is perhaps indicated by its more recent population decline. It is imperative that we understand the population dynamics of such a species at a much finer scale, which could be possible with sampling more populations, particularly those that may connect the study populations. What is of greater concern, however, is the clear genetic signature of a serious decline in the populations of the species, possibly mediated by the extensive hunting that it continues to face across its distribution range. While these threats to the affected populations are currently being documented by on-ground field studies, it is entirely possible that unless immediate action is taken, genetic drift caused by hunting, epidemics or other natural calamities can rapidly eliminate the remaining genetic diversity of this less-known endemic primate from an extremely important biodiversity hotspot in the remote Himalayan mountains of northeastern India.

## Supporting Information

Figure S1 **Map of Arunachal Pradesh, northeastern India, with locations of the sampling sites.** The triangles correspond to sampling sites in West Siang, circles to those in Upper Subansiri and rectangles to Tawang sampling sites. Inset: Location of the study site at the edge of Tibetan Plateau.
(TIF)

Table S1 **Arunachal and bonnet macaque samples used in the study and their sites of origin.**
(DOCX)
