# Correction: Mixed Fortunes: Ancient Expansion and Recent Decline in Population Size of a Subtropical Montane Primate, the Arunachal Macaque *Macaca munzala*

**DOI:** 10.1371/journal.pone.0116243

**Published:** 2014-12-17

**Authors:** The PLOS ONE Staff

The image for Figure S1 is incomplete. Please view the correct Figure S1 below.

## Supporting Information

Figure S1 **Map of Arunachal Pradesh, northeastern India, with locations of the sampling sites.** The triangles correspond to sampling sites in West Siang, circles to those in Upper Subansiri and rectangles to Tawang sampling sites. Inset: Location of the study site at the edge of Tibetan Plateau.
(TIF)
