# Supplementary figures and images for: Mixed Fortunes: Ancient Expansion and Recent Decline in Population Size of a Subtropical Montane Primate, the Arunachal Macaque Macaca munzala
Source: PLoS One. 2014 Jul 23;9(7):e97061. doi: 10.1371/journal.pone.0097061 (PMC4108313; doi:10.1371/journal.pone.0097061)

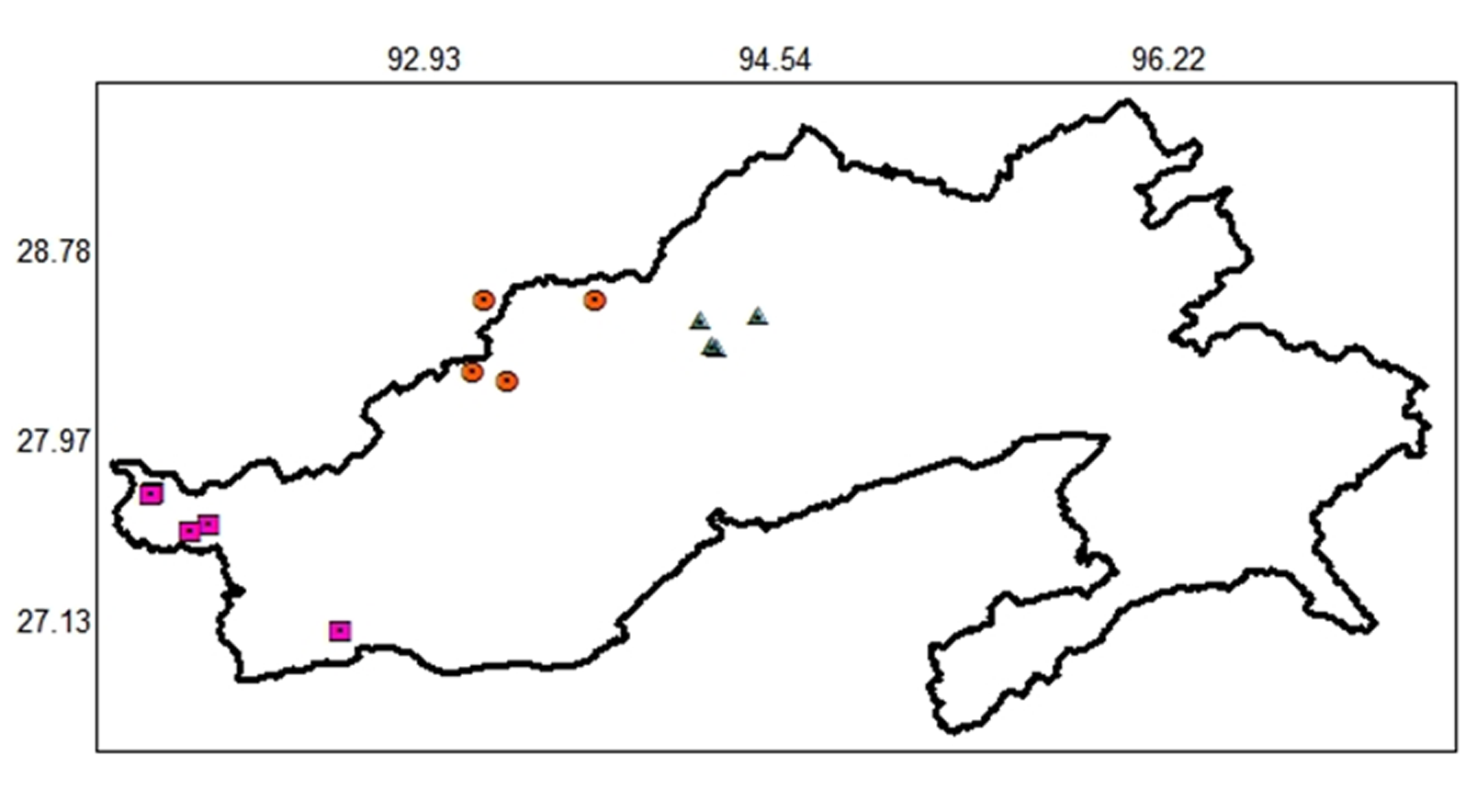

Supplement: Figure S1 — Map of Arunachal Pradesh, northeastern India, with locations of the sampling sites. The triangles correspond to sampling sites in West Siang, circles to those in Upper Subansiri and rectangles to Tawang sampling sites. Inset: Location of the study site at the edge of Tibetan Plateau. (TIF) [file pone.0097061.s001.tif]
