# Supplementary material for: Mixed Fortunes: Ancient Expansion and Recent Decline in Population Size of a Subtropical Montane Primate, the Arunachal Macaque Macaca munzala
Source: PLoS One. 2014 Jul 23;9(7):e97061. doi: 10.1371/journal.pone.0097061 (PMC4108313; doi:10.1371/journal.pone.0097061)
Supplement: Table S1 — Arunachal and bonnet macaque samples used in the study and their sites of origin. (DOCX) [file pone.0097061.s002.docx]

| **Species, Population** | **Location** | **Sample** | **Latitude** | **Longitude** | **Altitude (m)** | **Accession Numbers** |
| --- | --- | --- | --- | --- | --- | --- |
| Arunachal macaque, Tawang | Jang | S1 | 27.58 | 91.98 | - | KC844118 |
|  | Gronkhar | S2 | 27.55 | 91.90 | 1997 | KC844119 |
|  | Zemithang | S3 | 27.72 | 91.73 | - | KC844120 |
|  | Typee | S6 | 27.11 | 92.57 | - | KC844122 |
|  | Lomphu | S7 | 27.71 | 91.72 | - | KC844123 |
| Arunachal macaque, Upper Subansiri | Taksing | S4 | 28.58 | 93.22 | - | KC844121 |
|  | Ketenallah | S9 | 28.21 | 93.32 | 1133 | KC844125 |
|  | Yeaza | S10 | 28.26 | 93.16 | 2100 | KC844126 |
|  | Taksing | S11 | 28.58 | 93.72 | 2000 | KC844127 |
|  |  | S12 | 28.58 | 93.72 | 2000 | KC844128 |
|  |  | S13 | 28.58 | 93.72 | 2400 | KC844129 |
|  |  | S15 | 28.58 | 93.72 | 2100 | KC844130 |
|  |  | S16 | 28.58 | 93.72 | 2100 | KC844131 |
|  | TCC Camp | S17 | 28.58 | 93.72 | 2400 | KC844132 |
|  |  | S18 | 28.58 | 93.72 | 2000 | KC844133 |
| Arunachal macaque, West Siang | Lungte | S19 | 28.36 | 94.24 | 1480 | KC844134 |
|  |  | S20 | 28.36 | 94.24 | 1480 | KC844135 |
|  |  | S21 | 28.36 | 94.24 | 1480 | KC844136 |
|  | Peidi | S22 | 28.37 | 94.21 | 1640 | KC844137 |
|  | Papikurung | S23 | 28.48 | 94.16 | 2051 | KC844138 |
|  |  | S24 | 28.48 | 94.16 | 2051 | KC844139 |
|  |  | S25 | 28.48 | 94.16 | 2051 | KC844140 |
|  |  | S26 | 28.48 | 94.16 | 2051 | KC844141 |
|  | Tato Gitu | S40 | 28.51 | 94.42 | 1238 | KC844142 |
